# The INNODIA Type 1 Diabetes Natural History Study: a European cohort of newly diagnosed children, adolescents and adults

**DOI:** 10.1007/s00125-024-06124-5

**Published:** 2024-03-22

**Authors:** M. Loredana Marcovecchio, A. Emile J. Hendriks, Carl Delfin, Tadej Battelino, Thomas Danne, Mark L. Evans, Jesper Johannesen, Simranjeet Kaur, Mikael Knip, Lut Overbergh, Flemming Pociot, John A. Todd, Bart Van der Schueren, Linda S. Wicker, Mark Peakman, Chantal Mathieu, on behalf of the INNODIA consortium

**Affiliations:** 1 Department of Paediatrics, University of Cambridge, Cambridge, UK; 2 Department of Paediatric Diabetes and Endocrinology, Cambridge University Hospitals NHS Foundation Trust, Cambridge, UK; 3 Department of Pharmacometrics, Novo Nordisk A/S, Søborg, Denmark; 4 Department of Endocrinology, Diabetes and Metabolism, University Children’s Hospital, University Medical Centre Ljubljana, Ljubljana, Slovenia; 5 Faculty of Medicine, University of Ljubljana, Ljubljana, Slovenia; 6 Centre for Paediatric Endocrinology, Diabetology, and Clinical Research, Auf Der Bult Children’s Hospital, Hannover, Germany; 7 Wellcome MRC Institute of Metabolic Science, University of Cambridge, Cambridge, UK; 8 Department of Medicine, University of Cambridge, Cambridge, UK; 9 Translational Type 1 Diabetes Research, Clinical Research, Steno Diabetes Center Copenhagen, Herlev, Denmark; 10 Department of Paediatrics, Copenhagen University Hospital, Herlev, Denmark; Institute of Health and Medical Sciences, University of Copenhagen, Herlev, Denmark; 11 Research Program for Clinical and Molecular Metabolism, Faculty of Medicine, University of Helsinki, Helsinki, Finland; 12 Pediatric Research Center, New Children’s Hospital, Helsinki University Hospital, Helsinki, Finland; 13 Clinical and Experimental Endocrinology, Department of Chronic Diseases and Metabolism, KU Leuven, Leuven, Belgium; 14 Centre for Human Genetics, Nuffield Department of Medicine, University of Oxford, Oxford, UK; 15 Immunology & Inflammation Research Therapeutic Area, Sanofi, MA USA

**Keywords:** Age, Beta cell function, C-peptide, Prevention, Subgroups, Treatment, Type 1 diabetes

## Abstract

**Aims/hypothesis:**

Type 1 diabetes is an heterogenous condition. Characterising factors explaining differences in an individual’s clinical course and treatment response will have important clinical and research implications. Our aim was to explore type 1 diabetes heterogeneity, as assessed by clinical characteristics, autoantibodies, beta cell function and glycaemic outcomes, during the first 12 months from diagnosis, and how it relates to age at diagnosis.

**Methods:**

Data were collected from the large INNODIA cohort of individuals (aged 1.0–45.0 years) newly diagnosed with type 1 diabetes, followed 3 monthly, to assess clinical characteristics, C-peptide, HbA_1c_ and diabetes-associated antibodies, and their changes, during the first 12 months from diagnosis, across three age groups: <10 years; 10–17 years; and ≥18 years.

**Results:**

The study population included 649 individuals (57.3% male; age 12.1±8.3 years), 96.9% of whom were positive for one or more diabetes-related antibodies. Baseline (IQR) fasting C-peptide was 242.0 (139.0–382.0) pmol/l (AUC 749.3 [466.2–1106.1] pmol/l × min), with levels increasing with age (*p*<0.001). Over time, C-peptide remained lower in participants aged <10 years but it declined in all age groups. In parallel, glucose levels progressively increased. Lower baseline fasting C-peptide, BMI SD score and presence of diabetic ketoacidosis at diagnosis were associated with lower stimulated C-peptide over time. HbA_1c_ decreased during the first 3 months (*p*<0.001), whereas insulin requirement increased from 3 months post diagnosis (*p*<0.001).

**Conclusions/interpretation:**

In this large cohort with newly diagnosed type 1 diabetes, we identified age-related differences in clinical and biochemical variables. Of note, C-peptide was lower in younger children but there were no main age differences in its rate of decline.

**Graphical Abstract:**

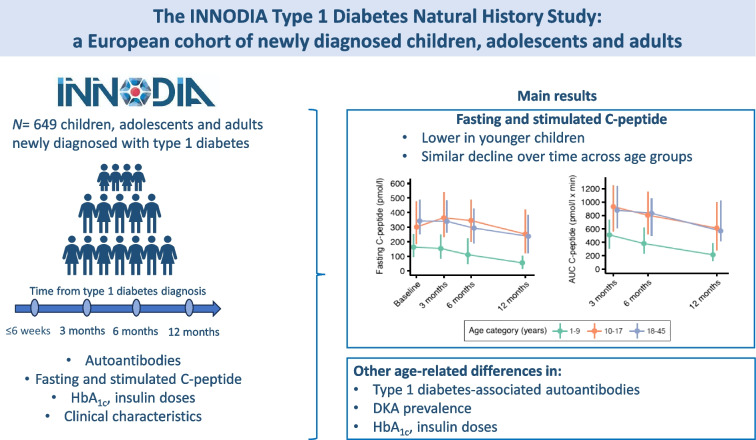

**Supplementary Information:**

The online version of this article (10.1007/s00125-024-06124-5) contains peer-reviewed but unedited supplementary material.


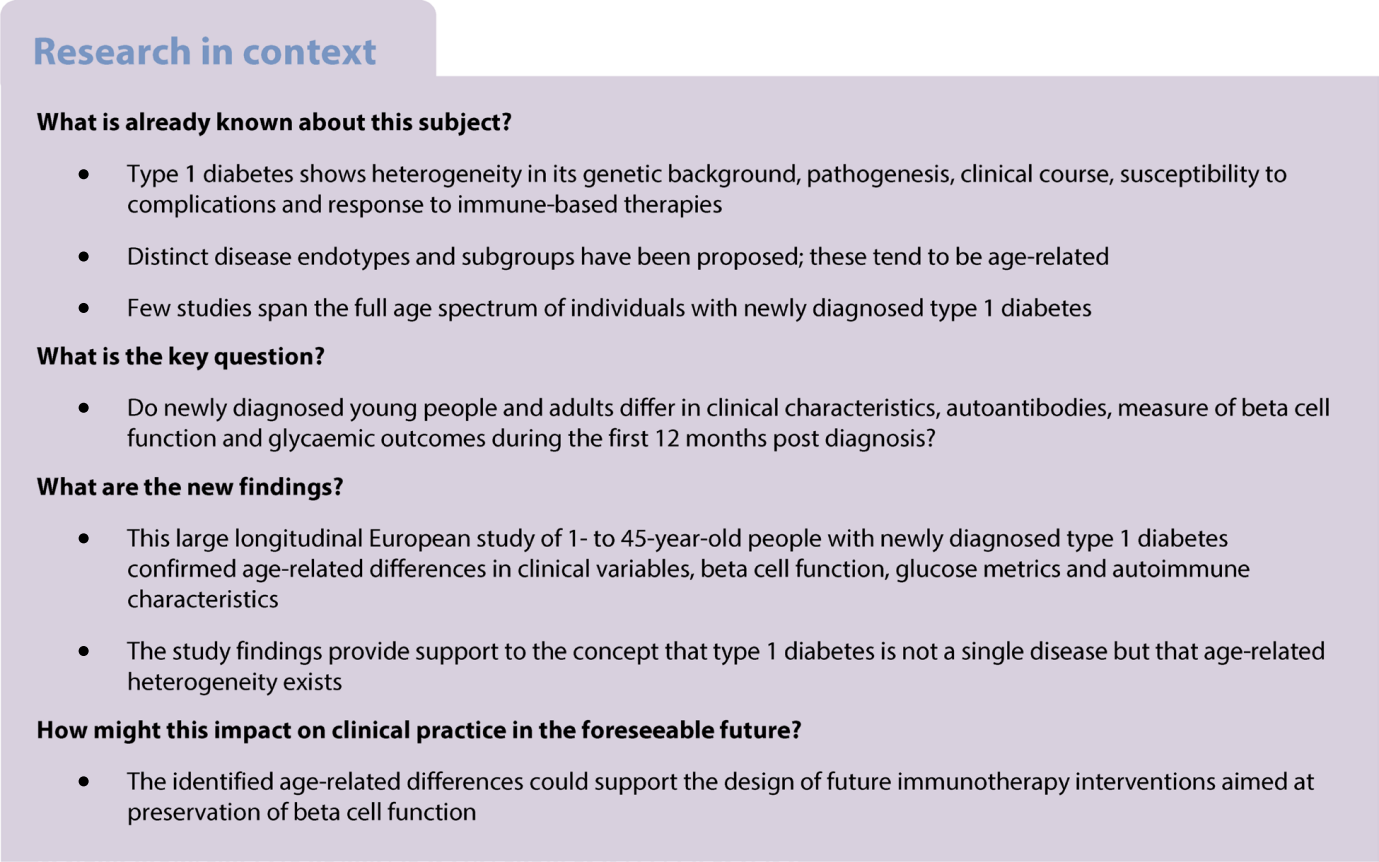


## Introduction

Type 1 diabetes results from an autoimmune response that leads to destruction of the pancreatic beta cells, consequent insulin deficiency and life-long need for exogenous insulin [1, 2].

Growing evidence supports the existence of heterogeneity in type 1 diabetes genetic background, pathogenesis, clinical course, susceptibility to complications and response to immune-based therapies [1, 3–5]. This has led to the concept that type 1 diabetes is not a single disease but that distinct subtypes (so called endotypes) exist, and that these subtypes tend to be age-related [1, 6–8]. Recognising such heterogeneity and a detailed characterisation of individuals’ subgroups could provide critical information for the design of future immunotherapy interventions aimed at arresting disease progression and moving towards precision medicine [1, 9].

Longitudinal cohorts of individuals newly diagnosed with type 1 diabetes represent an invaluable resource for characterising people close to diagnosis and gaining insights into the changes occurring in clinical and biochemical characteristics over time and how they might differ according to type 1 diabetes being diagnosed during childhood, adolescence or adulthood.

The Innovative approach towards understanding and arresting type 1 diabetes (INNODIA) consortium was established with the purpose of developing a European infrastructure for the recruitment, detailed clinical phenotyping and biosampling of a large cohort of newly diagnosed individuals with type 1 diabetes and unaffected family members using the INNODIA Master protocol [10]. The overall aim of INNODIA is to advance how to predict, stage, evaluate and prevent the onset and progression of type 1 diabetes.

Using the INNODIA infrastructure, we set out to explore the heterogeneity of type 1 diabetes, as assessed by clinical characteristics, autoantibodies, measures of beta cell function and glycaemic outcomes, and how it relates to age at diagnosis, in a large cohort of children, adolescents and adults during the first 12 months from diagnosis.

## Methods

### Study design

The INNODIA Natural History Study is a multicentre study involving 18 main diabetes clinical centres across Europe. These academic centres work with regional networks and there are 47 active clinical sites overall (https://www.innodia.eu/) [10].

The study protocol conformed to the Declaration of Helsinki and was initially approved by the London–City & East Research Ethics Committee (REC 16/lO/1750; IRAS Project ID 210497). Subsequently, after translation of the participants’ documentation, approval was obtained from other local Ethic authorities. Parents of participants provided written informed consent, and participants were asked to provide their assent, until they reached an age when they could consent themselves.

### Study population

Participants were identified through adult and paediatric diabetes clinics at participating sites and recruited between November 2016 and November 2021. Here, we report data collected up to October 2022. Inclusion criteria were as follows: (1) age 1–45 years; (2) diagnosed with type 1 diabetes within 6 weeks; and (3) written informed consent. Exclusion criteria were as follows: (1) non-type 1 diabetes; (2) use of long-term immunosuppressive agents including oral steroids or medications likely to confound the interpretation of study results; and (3) any other condition that might compromise study participation or confound interpretation of the results.

### Assessments

All participants had a baseline assessment within 6 weeks from diagnosis of type 1 diabetes (based on the ADA criteria [11], defined as the time at which insulin therapy was started), including collection of medical and family history, self-reported sex, and assessment of height, weight and BMI. Blood samples were collected for HbA_1c_, DNA extraction, type 1 diabetes-associated antibodies (GAD 65 autoantibodies [GAD65A], insulinoma-2 antigen autoantibodies [IA-2A], ZnT8A, antibodies to exogenous insulin [IA]/IAA), fasting C-peptide and peripheral blood mononuclear cells. Urine was collected for biomarker discovery and stool samples were collected for microbiome analysis.

### Follow-up

Participants had follow-up visits at 3, 6, 12 and 24 months. At each visit, height and weight were measured and BMI was calculated. Data on insulin doses over the previous 3 days were recorded. Blood, urine and stool samples were collected for the same assessments described for the baseline visit. Diabetes-associated antibodies were reassessed at 12 and 24 months only.

Participants aged ≥5 years had a mixed-meal tolerance test (MMTT) undertaken at each visit, with the first MMTT performed at the 3 month visit. Participants aged <5 years had only a fasting C-peptide assessed at any follow-up visit instead of the MMTT.

Information collected at each study visit was recorded into the electronic case report form (eCRF) in the INNODIA Data Warehouse [10].

### Laboratory methods

#### C-peptide, glucose and HbA_1c_

Fasting plasma C-peptide and serial C-peptide samples taken during MMTT were assayed in singleton on a DiaSorin Liaison XL automated immunoassay analyser with a sandwich chemiluminescence immunoassay (DiaSorin, Saluggia, Italy). Between-batch imprecision for the assay is 6.6% at 584 pmol/l, 5.6% at 2629 pmol/l and 5.4% at 5793 pmol/l (in-house data). HbA_1c_ and glucose were analysed using local laboratory methods using international standardisation.

#### Autoantibodies

Three autoantibodies, GAD65A, IA-2A and ZnT8A, were analysed in the PEDIA laboratory (University of Helsinki) and were quantified with the use of specific radiobinding assays as described earlier [12]. Insulin antibodies were also measured by a specific radiobinding assay [12]. Since the baseline sample was taken up to 6 weeks after the start of insulin treatment, and the method applied is unable to discriminate between IAA and IA, the baseline result represents an individual mix of IAA and IA depending on how soon the sample was collected after the initiation of insulin and the participant’s ability to mount a humoral immune response to exogenous insulin. The results from the samples taken during follow-up represent true IAs.

#### HLA

Genotyping of DNA extracted from peripheral blood was performed using Affymetrix UK Biobank Axiom Array (Affymetrix CytoScan 750k). SNP variant quality control was performed before imputation, and SNPs were filtered on SNP genotype missingness (<1%), Hardy–Weinberg equilibrium (*p*<1×10^−6^) and minor allele frequency (<1%). The genotyping data were then imputed to the HRC (GRCh37) reference panel. The imputation quality score (INFO>0.4) was used to filter poor quality SNPs. The highest risk heterozygous genotypes were identified based on the two SNPs (rs2187668 and rs7454108) that capture the *DR3/DR4-DQ8* haplotypes [13] and from imputed HLA alleles using SNP2HLA with T1DGC reference panel [14]. For HLA stratification, three groups were identified as follows: Group 1 (*DR3/DR4*; *DR4/DR4*; *DR3/DR3*); Group 2 (*DR3/DRX*, *DR4/DRX*); and Group 3 (*DRX*/*DRX*).

#### MMTT

A 2 h MMTT was performed under fasting conditions. Participants were instructed not to administer short-acting insulin within 6 h prior to the test. The test was only performed if the fasting glucose was between 4 and 11.1 mmol/l. Participants were given 6 ml/kg of Ensure Plus (Abbott Nutrition, UK) meal solution (up to a maximum of 360 ml) and blood samples for C-peptide and glucose measurements were collected 10 min prior to the meal (−10 min), at the time of ingestion (0 min) and at 15, 30, 60, 90 and 120 min thereafter.

### Calculations

Age- and sex-appropriate SD scores (SDS) were calculated for height, weight and BMI using WHO 2006/2007 data [15]. The AUC for glucose and C-peptide were computed using a trapezoidal rule, which is a weighted sum of the C-peptide values over 120 min. The insulin-dose-adjusted A1c (IDAA1c) was calculated as HbA_1c_ (%) + (4×insulin dose/kg) and an IDAA1c ≤9 was used to define partial remission, as previously reported [16].

### Statistical analyses

Descriptive summaries for baseline measurements are presented as median (IQR) or mean ± SD unless otherwise specified. Baseline characteristics were compared across three age groups (1–9.9 years [childhood], 10–17.9 years [adolescence] and 18–45 years adulthood]) using ANOVA for continuous variables and *χ*^2^ test for categorical/dichotomised variables. Tukey adjusted *p* values for pairwise post hoc *t* tests are reported following statistically significant main effects for continuous variables. False discovery rate (FDR)-adjusted *p* values for pairwise post hoc Fisher’s exact tests are reported following statistically significant main effects for categorical/dichotomised variables. Linear mixed-effects models, with a random intercept for each participant, were used to model the longitudinal trajectories of glucose metabolism variables across age groups during the first 12 months post diagnosis. For each outcome, four different models were tested: (1) an intercept-only model; (2) adding time; (3) adding age group and a time × age group interaction term; and (4) adding baseline characteristics as covariates (weeks from diagnosis, ethnicity [Europe vs non-Europe], sex, HLA group, BMI SDS, diabetic ketoacidosis [DKA], HbA_1c_, insulin dose, fasting glucose, fasting C-peptide, number of autoantibodies). Model fit was evaluated using *χ*^2^ tests. Type II Wald *F* tests with Kenward–Roger approximation for *df*- and FDR-adjusted *p* values were used to test for significant main effects for the best-fitting model for each outcome.

Statistical significance threshold was *p*≤0.05. All analyses were performed using R version 4.0.0 (R Project for Statistical Computing, R Core Team, Vienna, Austria).

## Results

**Fig. 1:**
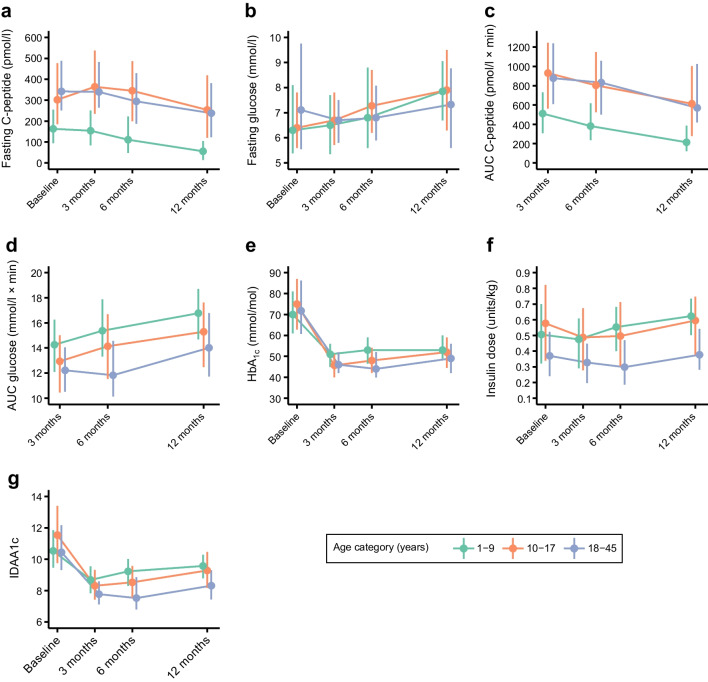
Time course of glucose metabolism variables during the first 12 months post diagnosis by age group. Data are shown as median and IQR for fasting C-peptide (**a**), fasting glucose (**b**), AUC C-peptide (**c**), AUC glucose (**d**), HbA_1c_ (**e**), insulin dose (**f**) and IDAA1c (**g**). Number of participants with completed data at baseline by age group (1–9; 10–17; 18–45 years): fasting C-peptide (246; 244; 92), fasting glucose (246; 244; 92), AUC C-peptide (134; 188; 69), AUC glucose (140; 205; 75), HbA_1c_ (249; 253; 92), insulin dose (264; 256; 93) and IDAA1c (242; 241; 86)

Six-hundred and seventy-three children, adolescents and young adults newly diagnosed with type 1 diabetes consented to the study. Fourteen withdrew before the first study visit and were excluded; an additional four had a missed baseline visit and six were later excluded following diagnosis reassessment due to lack of autoantibodies (four had a monogenic diabetes [genetically confirmed] and two had their diabetes reclassified as type 2) (electronic supplementary material [ESM] Fig. 1). Thus, the reported analysis is based on 649 participants with at least one study visit. Participants were recruited across 11 European countries, with the first baseline assessment taking place after a median of 4.9 (IQR 3.3–5.7) weeks from diagnosis.

Retention of participants in the study was 86% at 3 months, 82% at 6 months and 74% at 12 months.

### Clinical characteristics at baseline across age groups

Baseline characteristics of the study population are summarised in Table 1. Participants were grouped by age at diagnosis: 1.0–9.9 years (*n*=279, 43.0%), 10.0–17.9 years (*n*=270, 41.6%), 18.0–45.0 years (*n*=100, 15.4%). There were no significant differences in sex distribution across age groups. There were small differences in recruitment rates across countries due to only paediatric or adult sites being involved in some countries.

**Table 1:** Baseline clinical and biochemical characteristics by age

Characteristic	Overall	Age at baseline			*p* for trend	*p* value (1–9 vs 10–17 years)	*p* value (10–17 vs 18–45 years)	*p* value (1–9 vs 18–45 years)
		1–9 years	10–17 years	18–45 years				
Number	649	279 (43.0)	270 (41.6)	100 (15.4)				
Age at baseline, years	12.1±8.3	5.7±2.5	12.8±2.1	28.0±7.2				
Sex: male, *n* (%)	372 (57.3)	150 (53.8)	163 (60.4)	59 (59.0)	0.199			
Ethnicity, *n* (%)					<0.001	<0.001	<0.001	<0.001
European	524 (80.7)	214 (76.7)	221 (81.9)	89 (89.0)				
Asian	11 (1.7)	3 (1.1)	4 (1.5)	4 (4.0)				
African	21 (3.2)	9 (3.2)	10 (3.7)	2 (2.0)				
North American	2 (0.3)	1 (0.4)	1 (0.4)	0 (0.0)				
South American	2 (0.3)	1 (0.4)	0 (0.0)	1 (1.0)				
Mixed	17 (2.6)	7 (2.5)	9 (3.3)	1 (1.0)				
Not stated/available	72 (11.1)	44 (15.8)	25 (9.3)	3 (3.0)				
Country, *n* (%)					0.049	0.58	0.004	0.026
Austria	52 (8.0)	12 (4.3)	20 (7.4)	20 (20.0)				
Belgium	45 (6.9)	4 (1.4)	15 (5.6)	27 (27.0)				
Denmark	51 (7.9)	11 (3.9)	39 (14.4)	1 (1.0)				
Finland	195 (30.0)	124 (44.4)	69 (25.6)	2 (2.0)				
Germany	22 (3.4)	11 (3.9)	4 (1.5)	7 (7.0)				
Italy	65 (10.0)	21 (7.5)	32 (11.9)	12 (12.0)				
Luxembourg	37 (5.7)	18 (6.5)	15 (5.6)	3 (3.0)				
Poland	48 (7.4)	26 (9.3)	20 (7.4)	2 (2.0)				
Slovenia	50 (7.7)	20 (7.2)	30 (11.1)	0 (0.0)				
UK	84 (12.9)	32 (11.5)	26 (9.6)	26 (6.0)				
HLA, *n* (%)					0.190			
*DR3/DR3*	38 (6.7)	12 (5.1)	15 (6.3)	11 (12.0)				
*DR3/DR4*	121 (21.5)	57 (24.3)	49 (20.7)	15 (16.3)				
*DR3/DRX*	95 (16.8)	38 (16.2)	44 (18.6)	13 (14.1)				
*DR4/DR4*	29 (5.1)	13 (5.5)	10 (4.2)	6 (6.5)				
*DR4/DRX*	187 (33.2)	80 (34.0)	82 (34.6)	25 (27.2)				
*DRX/DRX*	94 (16.7)	35 (14.9)	37 (15.6)	22 (23.9)				
HLA group, *n* (%)^a,b^					0.165			
Group 1	188 (33.3)	82 (34.9)	74 (31.2)	32 (34.7)				
Group 2	282 (50.0)	118 (50.2)	126 (53.1)	38 (41.3)				
Group 3	94 (16.7)	35 (14.9)	37 (15.6)	22 (23.9)				
Height, m	1.45±0.28	1.18±0.18	1.61±0.13	1.76±0.10	<0.001	<0.001	<0.001	<0.001
Weight, kg	43.2±22.4	24.1±8.6	51.5±14.0	74.0±19.1	<0.001	<0.001	<0.001	<0.001
BMI at baseline, kg/m^2^	19.1±4.3	16.7±2.3	19.7±3.5	23.9±5.5	<0.001	<0.001	<0.001	<0.001
BMI SDS	0.32±1.65	0.36±1.86	0.17±1.38	0.65±1.68	0.042	0.355	0.302	0.037
Duration of type 1 diabetes, weeks	4.9 (3.3–5.7)	4.7 (3.1–5.9)	4.9 (3.4–5.7)	4.9 (3.6–5.7)	0.21			
DKA at diagnosis, *n* (%)	233 (36)	91 (32.8)	119 (43.9)	23 (23.0)	<0.001	0.027	0.058	0.001
HbA_1c_ at diagnosis, mmol/mol	103.0 (84.8–122.0)	95.0 (76.0–114.0)	110 (95.0–128.3)	99.0 (82.3–124.8)	<0.001	<0.001	0.217	0.06
HbA_1c_ at diagnosis, %	11.6 (9.9–13.3)	10.8 (9.1–12.6)	12.2 (10.8–13.9)	11.2 (9.7–13.6)	<0.001	<0.001	0.217	0.06
HbA_1c_ at baseline, mmol/mol	72.7 (61.0–83.6)	70.0 (61.0–81.0)	75.0 (62.9–87.0)	71.8 (60.8–86.2)	0.002	0.003	0.527	0.373
HbA_1c_ at baseline, %	8.8 (7.7 −9.8)	8.6 (7.7–9.6)	9.0 (7.9–10.1)	8.7 (7.7–10.0)	0.002	0.003	0.527	0.373
Insulin dose (U/kg per day)	0.55±0.34	0.55±0.32	0.62±0.38	0.40±0.23	<0.001	0.053	0.001	<0.001
IDAA1c	11.2±2.6	10.9±2.3	11.7±2.9	10.7±2.1	<0.001	0.001	0.83	0.004
Fasting glucose, mmol/l	8.9±13.4	9.5±17.8	7.9±7.3	9.9±11.6	0.309			
Fasting C-peptide, pmol/l	242.0 (139.0–382.0)	163.0 (94.8–254.8)	299.5 (184.8–477.5)	342.0 (251.5–488.0)	<0.001	<0.001	<0.001	0.015
AUC C-peptide, pmol/l × min	749.3 (466.2–1106.1)	511.4 (309.6–730.1)	956.2 (591.0–1254.5)	887.3 (609.8–1240.9)	<0.001	0.014	0.012	0.675
AUC glucose, mmol/l × min	13.2±3.1	14.2±3.1	12.8±3.1	12.6±2.9	<0.001	<0.001	0.001	0.867

Data are presented as *n* (%), mean ± SD or median (IQR)

^a^HLA groups: group 1, *DR3/DR4*, *DR4/DR4*, *DR3/DR3*; group 2, *DR3/DRX*, *DR4/DRX*; group 3, *DRX/DRX*

^b^HLA results are based on 564 participants with available data at the time of the analysis

Overall, the prevalence of DKA at diagnosis was 36%, with the highest rate in the group aged 10–17 years (44%) and the lowest rate in the group aged 18–45 years (23%). Mean BMI SDS at baseline was 0.32, with the lowest BMI SDS in the 10–17 years old group. Median HbA_1c_ at diagnosis was 103.0 (IQR 84.8–122.0) mmol/mol (11.6 [9.9–13.3]%), with the highest values in the 10–17 years age group. At baseline, median HbA_1c_ levels decreased to 72.7 (IQR 61.0–83.6) mmol/mol (8.8 [7.7–9.8]%). Baseline total daily insulin dose was 0.55±0.34 U/kg, with the lowest dose in the ≥18 years old group. The mean ± SD IDAA1c was 11.2±2.6, with the highest value in the age group 10–17 years old.

Median (IQR) C-peptide at baseline was 242.0 (139.0–382.0) pmol/l, with levels progressively increasing across age groups: <10 years 163.0 (94.8–254.8) pmol/l; 10–17 years 299.5 (184.8–477.5) pmol/l; and 18–45 years 342.0 (251.5–488.0) pmol/l (*p*<0.001).

For HLA risk as defined by Groups 1–3, there was no statistically significant difference between age groups (Table 1).

### Islet antibody positivity at baseline

Twenty participants (3.1%) tested negative for all diabetes-associated antibodies at baseline, with a progressive increase in the proportion of antibody-negative participants from the youngest to the oldest age group (Table 2). When excluding IAA/IA, the proportion of participants who tested negative for the three autoantibodies increased to 6%.

**Table 2:** Diabetes-associated antibody status at baseline and 12-month follow-up

No. of antibodies	Overall	1–9 years	10–17 years	18–45 years
Antibody positive at baseline, *n* (%)				
0	20 (3.1)	3 (1.1)	9 (3.3)	8 (8.0)
1	49 (7.5)	13 (4.7)	22 (8.1)	14 (14.0)
2	132 (20.2)	71 (25.5)	37 (13.7)	24 (24.0)
3	220 (33.6)	84 (30.2)	104 (38.4)	32 (32.0)
4	214 (32.7)	103 (37.1)	96 (35.4)	15 (15.0)
Antibody positive at 12 months, *n* (%)				
0	3 (0.5)	1 (0.6)	0 (0.0)	3 (3.8)
1	37 (5.7)	17 (9.7)	11 (5.0)	9 (11.5)
2	91 (13.9)	42 (24.0)	40 (18.3)	9 (11.5)
3	145 (22.2)	56 (32.0)	54 (24.7)	35 (44.9)
4	157 (24.0)	43 (24.6)	94 (42.9)	20 (25.6)

The order of detection of autoantibodies was IAA/IA (78%), GAD65A (74%), IA-2A (70%) and then ZnT8A (67%) (ESM Table 1). GAD65A was the most frequent autoantibody type in participants older than 18 years (78%), whereas IAA/IA (87%) and IA-2A (76%) were the predominant autoantibodies in those younger than 10 years, and GAD65A (78%) and IAA/IA (78%) in those 10–17 years-old.

### Time course of glucose metabolism variables during the first 12 months post diagnosis

Fasting C-peptide showed a progressive decline during the first 12 months post diagnosis, particularly from 3 months onwards (Fig. 1a). A similar decline was observed across the three age groups and overall levels remained lower in the youngest age group (1–9 years old). The decline in fasting C-peptide was associated with a progressive increase in fasting glucose (Fig. 1b).

The AUC for C-peptide during the MMTT showed a decline over time in all three age groups, with values consistently lower in participants aged 1–9 years (Fig. 1c). The trend in AUC for glucose during the MMTT (Fig. 1d) was similar to that for fasting glucose.

**Fig. 2:**
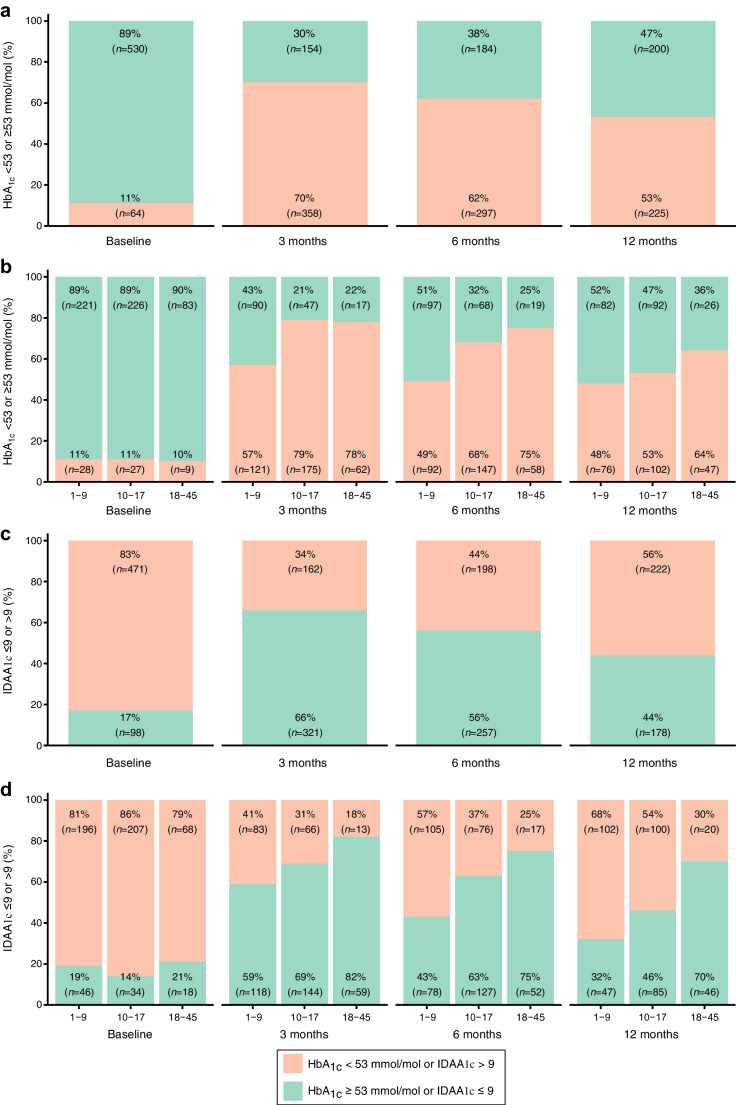
Participants with HbA_1c_ <53 mmol/mol and IDAA1c ≤9 at baseline and follow-up. (**a**, **b**) Proportion of all study participants with HbA_1c_ <53 mmol/mol (orange bars) or HbA_1c_ ≥53 mmol/mol (green bars) at baseline and follow-up visits (3, 6, 12 months) (**a**), and divided by age groups (1–9, 10–17 and 18–45 years) (**b**). (**c**, **d**) Proportion of all participants with IDAA1c ≤9 (green bars) or IDAA1c >9 (orange bars) at baseline and follow-up visits (3, 6, 12 months) (**c**) and divided by age groups (1–9, 10–17 and 18–45 years) (**d**)

HbA_1c_ decreased significantly during the first 3 months post diagnosis (Fig. 1e). The proportion of participants with an HbA_1c_<53 mmol/l (<7%) was 11% at baseline and increased to 53% at 12 months (Fig. 2a,b). There were small changes in insulin requirements over time, with a decrease during the first 3–6 months and a later increase at 12 months (Fig. 1f). IDAA1c mirrored the patterns in HbA_1c_ and insulin requirement (Fig. 1g), with an increase in the proportion of participants with values ≤9 during the first 3 months (from 17% to 66%) and then a gradual decrease (44% at 12 months) in all age groups (Fig 2c). The proportion of participants with an IDAA1c ≤9 from 3 months post diagnosis onwards was consistently lower in the younger age groups compared with the group of participants aged ≥18 years (Fig. 2d).

There were no differences between male and female participants (ESM Fig. 2) or across HLA risk groups (ESM Fig. 3), in baseline and changes over time, for C-peptide, glucose, HbA_1c_, insulin dose and IDAA1c.

To further assess the time course of glucose metabolism variables and the potential effect of baseline variables, a multivariate linear mixed model analysis was performed including time (months of follow-up), age groups, time × age group interaction and baseline clinical characteristics. In this model, the age group category 10–17 years old was significantly associated with higher AUC C-peptide, HbA_1c_ and IDAA1c, and non-significantly associated with higher fasting C-peptide (*p*=0.08) compared with the group aged 1–9 years old (Table 3). When considering the age × time interaction, fasting C-peptide was significantly higher in the 10–17 years group than in the 1–9 years group at each time point (3, 6 and 12 months) compared with baseline. Differences were also found for HbA_1c_, insulin and IDAA1c: all significantly lower in the 10–17 years old group. HbA_1c_ was also lower in the 18–45 years group than the 1–9 years group at each follow-up visit compared with baseline. In contrast, there were no significant differences in AUC C-peptide when comparing age groups at 6 and 12 months vs 3 months (time of first MMTT).

**Table 3:** Parameter estimates from multivariable mixed-effects models of baseline characteristics associated with glucose metabolism variables over time

Covariate	Fasting C-peptide		Fasting glucose		AUC C-peptide^a^		AUC fasting glucose^a^		HbA_1c_		Insulin dose		IDAA1c	
	Estimate	*P*_FDR_	Estimate	*P*_FDR_	Estimate	*P*_FDR_	Estimate	*P*_FDR_	Estimate	*P*_FDR_	Estimate	*P*_FDR_	Estimate	*P*_FDR_
Intercept	74.376	0.173	1.381	0.352	596.919	0.004	11.038	<0.001	39.465	<0.001	0.120	0.085	6.035	<0.001
Time (baseline as reference)														
3 months	−14.994	0.469	−1.838	<0.001	-	-	-	-	−19.513	<0.001	−0.081	<0.001	−2.099	<0.001
6 months	−34.939	0.059	−1.373	0.004	−80.054	0.020	1.344	<0.001	−19.233	<0.001	−0.009	0.791	−1.779	<0.001
12 months	−85.082	<0.001	−0.579	0.404	−245.174	<0.001	2.470	<0.001	−17.398	<0.001	0.076	<0.001	−1.285	<0.001
Age category (1–9 years as reference)														
10–17 years	35.449	0.083	−0.107	0.859	322.995	<0.001	−1.269	0.011	4.845	<0.001	0.040	0.173	0.593	<0.001
18–45 years	40.231	0.169	0.360	0.688	128.407	0.168	−0.634	0.451	1.151	0.688	−0.057	0.168	−0.113	0.780
Time × age category														
3 months, 10–17 years	97.208	<0.001	1.288	0.034	-	-	-	-	**−**12.544	<0.001	−0.074	0.034	−1.485	<0.001
6 months, 10–17 years	78.929	<0.001	1.592	0.011	−19.974	0.767	0.116	0.859	**−**11.114	<0.001	−0.120	<0.001	−1.519	<0.001
12 months, 10–17 years	73.231	0.007	1.072	0.169	−58.173	0.356	−0.283	0.121	−9.952	<0.001	−0.132	<0.001	−1.509	<0.001
3 months, 18–45 years	−11.499	0.811	−0.723	0.463	-	-	-	-	−5.582	0.046	0.048	0.414	−0.318	0.487
6 months, 18–45 years	−11.330	0.823	−0.882	0.404	−2.358	0.966	−1.117	0.129	−6.249	0.026	−0.004	0.957	−0.590	0.153
12 months, 18–45 years	−10.002	0.859	−1.008	0.404	68.430	0.414	−0.530	0.550	−5.952	0.048	−0.029	0.688	−0.630	0.150
Duration (weeks)	−5.803	0.184	0.098	0.420	−25.329	0.169	0.170	0.180	0.688	0.004	0.022	<0.001	0.153	<0.001
BMI SDS	10.989	0.017	0.106	0.451	51.028	<0.001	−0.209	0.090	0.133	0.724	−0.003	0.688	−0.008	0.859
DKA (absent as ref)	−24.994	0.090	−0.006	0.982	−132.633	0.017	0.309	0.532	−0.430	0.724	0.004	0.859	−0.035	0.859
HbA_1c_ (mmol/mol)	0.097	0.859	−0.001	0.957	−0.332	0.875	0.018	0.169	0.424	<0.001	0.000	0.670	0.039	<0.001
Insulin dose (U/kg)	−5.229	0.859	−0.355	0.577	18.131	0.859	1.082	0.063	−1.518	0.279	0.581	<0.001	2.223	<0.001
Fasting glucose (mmol/l)	−0.049	0.961	0.767	<0.001	−5.233	0.428	0.022	0.506	−0.004	0.954	0.001	0.624	0.004	0.724
Fasting C-peptide (pmol/l)	0.776	<0.001	0.000	0.724	1.326	<0.001	−0.002	0.007	−0.001	0.715	0.000	0.550	0.000	0.465
No. of autoantibodies	−6.139	0.420	0.113	0.546	−58.491	0.014	0.283	0.121	0.172	0.744	0.015	0.061	0.084	0.156

The models that included all variables under consideration (time, age group, time × age group interaction and the 11 baseline characteristics) showed superior model fit across all outcomes (all *p*<0.001). The model shows the effect (estimate) of baseline variables, age and time on changes in glycaemic outcomes over time. Other variables included in the model but not significantly associated with any outcome were sex, HLA group, ethnicity (European vs non-European)

^a^No data available at baseline (3 months used as reference)

*P*_FDR_, FDR-adjusted *p* value; statistically significant (*P*_FDR_ <0.05)

Among the baseline covariates affecting glucose metabolism variables over time, DKA at diagnosis was associated with lower AUC C-peptide (*p*<0.001). Lower BMI SDS was associated with higher fasting (*p*=0.017) and AUC C-peptide (*p*<0.001) over time. A higher number of autoantibodies at baseline was associated with lower AUC C-peptide over time. Fasting C-peptide at baseline was associated with higher fasting and AUC C-peptide and lower AUC glucose over time. Duration of diabetes at baseline was positively associated with HbA_1c_, insulin dose and IDAA1c levels.

### Diabetes-associated antibodies: changes between baseline and 12 months

There were no significant changes in antibody positivity for IA-2A (*p*=0.482) or GAD65A (*p*=0.157) between baseline and 12 months post diagnosis. ZnT8A showed a small decrease, from 67% to 61% positive (*p*=0.049), whereas IAA/IA showed a strong increase, from 78% to 98% positive (*p*<0.001) (Table 2 and ESM Table 1).

## Discussion

This study reports clinical and biochemical outcomes during the first 12 months from the clinical diagnosis of type 1 diabetes in a large European cohort of children, adolescents and adults recruited within 6 weeks of diagnosis.

The INNODIA cohort covers a wide age range, from 1 up to 45 years, providing a unique opportunity to identify potential age-related differences. Previous studies have highlighted the heterogeneity of type 1 diabetes and introduced the concept of ‘endotypes’, representing subtypes defined by distinct pathophysiological mechanisms [3, 4, 6–8]. Of interest these endotypes appear to be strongly associated with age at clinical diagnosis [3, 6–8]. Significant differences in islet pathology and genetic susceptibility were previously identified between children diagnosed before the age of 7 years vs ≥13 years [5, 7, 8].

In the present study, age groups were defined using different cutoffs to identify three main lifetime periods, namely childhood, adolescence and adulthood; these cutoffs resembled those used in some previous studies [17–19]. In our cohort, although overall there was a slightly higher prevalence of male participants (57.4%), confirming findings from previous studies [6, 17, 20], there were no significant differences in sex distribution across the three age groups. The prevalence of DKA at diagnosis was around 36% and was particularly high (44%) among adolescents. Recruitment to the INNODIA study started before the COVID-19 pandemic and continued during the pandemic. Therefore, the high rates of DKA likely reflect the reported higher prevalence of this acute complication at the time of clinical type 1 diabetes manifestation during the pandemic [21–24]. A recent multicentre study in 104,290 children and young people clearly showed that the prevalence of DKA at type 1 diabetes presentation increased from 27% during 2006–2019 to around 39% in 2020–2021, percentages that were higher than the predicted yearly rise [22]. These findings highlighted how the pandemic exacerbated an already increasing trend in DKA prevalence, likely due to delays in seeking medical attention, due to restrictions in place, and fear of contracting COVID-19 infection. The role of additional factors, such as a potential direct beta cell damage due to SARS-CoV-2, or the more general role of a viral infection in triggering clinically manifested type 1 diabetes in susceptible individuals should also be considered [25].

Although recent data confirm that a diagnosis of type 1 diabetes during early childhood is generally associated with the highest prevalence of DKA [26], in the INNODIA cohort DKA was particularly frequent among adolescents. This might reflect more severe presentations in this age group and longer duration of symptoms before seeking medical advice, often due to the reluctance of adolescents to bring their symptoms to parental attention. The high rates of DKA in the INNODIA cohort reinforce previous findings and highlight the need for further efforts to improve recognition of the presenting signs/symptoms by individuals, caregivers and healthcare professional by awareness campaigns [27]. Our results also support the ongoing discussion on the value of population screening for type 1 diabetes, associated with ad hoc education, continuous follow-up and support for individuals identified at risk and their families [28].

Of note, the present study showed age-related differences in C-peptide. Fasting and stimulated C-peptide levels were lowest in children younger than 10 years old, both at baseline and during the first 12 months post diagnosis. These age-related differences are similar to those reported in other multicentre studies such as TrialNet and Hvidoere, as well as national studies [17, 29, 30]. This finding might reflect a more aggressive disease pathogenesis/higher genetic risk or the role of other environmental factors in younger children [5, 29, 31–34]. It is, however, interesting to see that the evolution of C-peptide over the first 12 months of follow-up in the INNODIA cohort was similar in all age groups. Previous studies have either reported a more marked decline in younger children or no differences between age groups; these discrepancies might relate to duration of follow-up as well as differences in age distribution/age groups between studies [17, 19, 34]. As expected, and in line with previous data [17, 29, 32, 35], there was a progressive decline over time in C-peptide, which was affected by the presence of DKA and a lower BMI at baseline. DKA at the time of diagnosis was previously found to be associated with lower residual beta cell function over time [29], whereas data on the effect of BMI are discordant between previous studies [34, 36, 37].

The clinical benefits of preserving C-peptide in individuals newly diagnosed with type 1 diabetes are well known [38–40]. Residual beta cell function has been associated with better glycaemic outcomes and reduced complications risk [38, 40].

Along with changes in C-peptide, this study provides information on temporal changes in glycaemic metrics. HbA_1c_ levels, which at baseline were particularly high in the adolescent group, fell substantially during the first 3 months following diagnosis, in line with findings from previous studies [29, 35]. Only 47% of study participants achieved the recommended HbA_1c_ target of <53 mmol/mol (7%) [41] at 12 months post diagnosis.

Overall insulin requirement during the 12 months of follow-up was relatively low in the study population. This likely reflects the honeymoon phase and residual beta cell production. Insulin requirement was higher in children and adolescents than in adults and this might reflect differences in residual beta cell function as shown by C-peptide levels. The high doses in the age group 10–17 years old might also reflect pubertal insulin resistance [42] and/or more severe presentation. Indeed, this group showed higher prevalence of DKA at diagnosis as well as a lower BMI SDS, likely reflecting greater weight loss related to insulin deficiency and related metabolic abnormalities.

Trends in HbA_1c_ and insulin requirements were mirrored by the IDAA1c index, with a higher proportion of participants being in partial remission at 3 and 6 months post diagnosis, particularly among those older than 18 years.

Overall, a combination of IAA/IA, GAD65A, IA-2A and ZnT8A was found in around 97% of the study participants, with the highest frequency in children younger than 10 years old. As expected [43, 44], GAD65A were the most common autoantibody type in those aged ≥18 years, whereas IA-2A were the most frequent type in younger children. The prevalence of most autoantibodies remained unchanged when reassessed at 12 months, apart from a higher prevalence of IA, as expected following insulin therapy [29].

There were no HLA differences between age groups, in contrast with the findings of some previous studies [5]. This could be due to a lower sample size in the INNODIA cohort, limiting the ability to detect significant differences. However, the previously reported shift to fewer HLA-high risk genotypes in newly diagnosed type 1 diabetes [45, 46] could also explain our results.

Recruitment to the study was excellent and followed a stable pattern over time. Retention of participants was also very good, being around 74% at 12 months. These positive conclusions on recruitment and retention are even stronger when considering that the study was conducted during the COVID-19 pandemic.

Overall, the major study strength was the availability of a large cohort, including children, adolescents and adults, assessed very close to the clinical diagnosis of type 1 diabetes, and with data allowing the evaluation of early changes in clinical and biochemical variables during the first 12 months post diagnosis. INNODIA is a large European consortium with a particular interest in type 1 diabetes, based on a collaboration not only between academia and pharma but also with a strong contribution from people living with type 1 diabetes, represented by the INNODIA Patient Advisory Committee (PAC) [10]. This advisory committee reviewed and commented on study protocols and specific documents and constantly interacted with the INNODIA investigators to provide their views and suggestions to improve study design and its acceptability and feasibility.

This study provides data collected across many European centres following standardised procedures for data collection and processing, as per the INNODIA Master protocol [10]. Analysis of the major study endpoints (C-peptide, autoantibodies) were performed in centralised validated laboratories.

As with any large multicentre longitudinal study, there are limitations in the original design and choice of samples to be collected. One study limitation was lack of an MMTT within 6 weeks of diagnosis. However, in most previous clinical trials the first MMTT was performed within 100 days of diagnosis rather than anywhere close to 6 weeks. Data on MMTT were available for only 50% of the younger age group and around 70% of the older age groups. Differences in sample size across age groups, with a lower number of participants older than 18 years, and a potential floor effect for C-peptide levels in younger children could also have affected the study findings. Lack of ethnic heterogeneity, with most study participants being white European, limits the generalisability of the findings to other ethnic groups. However, the ethnic composition of the INNODIA cohort mirrors the ethnic characteristics of people with type 1 diabetes in Europe [29]. Another limitation is the lack of serum samples collected within 2 weeks after the start of insulin treatment, excluding the possibility of assessing the true frequency of IAA.

### Conclusions

Our study confirms that age-related differences in demographics, clinical features, beta cell function, glucose variables and autoimmune characteristics can be identified soon after diagnosis of type 1 diabetes and that these differences persist over time. Further understanding of the course of beta cell destruction in these age groups is essential to inform the design of future trials aimed at halting type 1 diabetes progression.

### Supplementary Information

Below is the link to the electronic supplementary material.

Supplementary file1 (PDF 323 KB)

## Data Availability

The data generated and analysed are person-sensitive and can be accessed in secure environments only. Access can be provided by application to the INNODIA Data Access Committee (innodia.eu).

## Abbreviations

DKA: Diabetic ketoacidosis
FDR: False discovery rate
GAD65A: GAD 65 autoantibodies
IDAA1c: Insulin-dose-adjusted A1c
IA: Antibodies to exogenous insulin
IA-2A: Insulinoma-2 antigen autoantibodies
INNODIA: Innovative approach towards understanding and arresting type 1 diabetes
MMTT: Mixed-meal tolerance test
SDS: SD score

## Members of the INNODIA and INNODIA HARVEST consortia

C. Mathieu, P. Gillard, K. Casteels, L. Overbergh (KU Leuven, Belgium); D. Dunger, C. Wallace, M. Evans, A. Thankamony, E. Hendriks, S. Bruggraber, M. L. Marcovecchio, Paediatrics laboratory staff (University of Cambridge, UK); M. Peakman, T. Tree (King’s College London, UK); N. Morgan, S. Richardson (University of Exeter, UK); J. Todd, L. Wicker (University of Oxford, UK); A. Mander, C. Dayan, M. Alhadj Ali (Cardiff University, UK); T. Pieber (Medical University of Graz, Austria); D. Eizirik, M. Cnop (Universite Libre de Bruxelles, Belgium); S. Brunak (University of Copenhagen, Denmark); F. Pociot, J. Johannesen, P. Rossing, C. Legido Quigley (Herlev University Hospital, Region Hovedstaden, Denmark); R. Mallone, R. Scharfmann, C. Boitard (Cochin Institute Paris, France); M. Knip, T. Otonkoski (University of Helsinki, Finland); R. Veijola (University of Oulu, Finland); R. Lahesmaa, M. Oresic, J. Toppari (University of Turku, Finland); T. Danne (Children’s and Youth Hospital Hannover, Germany); A. G. Ziegler, P. Achenbach, T. Rodriguez-Calvo (Helmholtz Zentrum Muenchen, Germany); M. Solimena, E. Bonifacio, S. Speier (TU Dresden, Germany); R. Holl (University of Ulm, Germany); F. Dotta (University of Siena, Italy); F. Chiarelli (University of Chieti, Italy); P. Marchetti (University of Pisa, Italy); E. Bosi (University Vita-Salute San Raffaele, Italy); S. Cianfarani, P. Ciampalini (Bambino Gesù Children’s Hospital, Italy); C. de Beaufort (Centre Hospitalier de Luxembourg, Luxemburg); K. Dahl-Jørgensen, T. Skrivarhaug, G. Joner, L. Krogvold (Oslo University Hospital, Norway); P. Jarosz-Chobot (Medical University of Silesia, Poland); T. Battelino, D. Smigoc Schweiger (University of Ljubljana, Slovenia); B. Thorens (University of Lausanne, Switzerland); M. Gotthardt (Radboud University Medical Center, the Netherlands); B. Roep, T. Nikolic, A. Zaldumbide (Leiden University Medical Center, the Netherlands); A. Lernmark, M. Lundgren (Lund University, Sweden); G. Costeca (Univercell-Biosolutions, France); T. Strube, A. Schulte, A. Nitsche (Sanofi, Germany); M. Peakman, J. Vela (Sanofi, USA); M. von Herrath, J. Wesley (Novo Nordisk, Denmark); A. Napolitano-Rosen (GlaxoSmithKline, UK); M. Thomas, N. Schloot (Eli Lilly, UK); A. Goldfine, F. Waldron-Lynch, J. Kompa, A. Vedala, N. Hartmann, G. Nicolas (Novartis Pharma AG, Switzerland); J. van Rampelbergh, N. Bovy (Imcyse SA, Belgium); S. Dutta, J. Soderberg, S. Ahmed, F. Martin, E. Latres (JDRF, USA); G. Agiostratidou, A. Koralova (The Leona M. and Harry B. Helmsley Charitable Trust, USA).

**Associated clinical sites**

R. Willemsen (Barts Health NHS Trust, UK); A. Smith (Northampton General Hospital NHS Trust, UK); B. Anand (West Suffolk NHS FT, UK); V. Puthi (North West Anglia NHS FT, UK); S. Zac-Varghese (East & North Hertfordshire NHS Trust, UK); V. Datta (Norfolk & Norwich University NHS FT, UK); R. Dias (Birmingham Women's and Children’s NHS FT, UK); P. Sundaram (University Hospitals of Leicester NHS Trust, UK); B. Vaidya (Royal Devon & Exeter NHS FT, UK); C. Patterson (NHS Fife, UK); K. Owen (Oxford University Hospitals NHS FT, UK); C. Dayan (Cardiff & Vale University Health Board, UK); B. Piel (Queen Elizabeth Hospital, King’s Lynn FT, UK); S. Heller (Sheffield Teaching Hospitals NHS FT, UK); T. Randell, T. Gazis (Nottingham University Hospitals NHS Trust, UK); E. Bismuth Reismen, J.-C. Carel (Hospital Robert Debre, France); J.-P. Riveline, J.-F. Gautier (Hospital Lariboisiere, France); F. Andreelli (Hospital Lapitie-Salpetriere, France); F. Travert (Hospital Bichat Claude Bernard, France); E. Cosson (Hospital Jean-Verdier, France); A. Penfornis, C. Petit (Centre Hospitalier Sud-Francilien, France); B. Feve (Hospital St Antoine, France); N. Lucidarme (Hospital Jean-Verdier Pediatrie, France); E. Cosson (Hospital Avicenne, France); J.-P. Beressi (Hospital Andre Mignot, France); C. Ajzenman (Hospital Andre Mignot Pediatrie, France); A. Radu (Hospital Europeen Georges-Pompidou, France); S. Greteau-Hamoumou (Hospital Louis Mourier, France); C. Bibal (Hospital Kremlin Bicetre, France); T. Meissner (Universitatsklinikum der Heinrich-Heine-Univeritat Dusseldorf, Germany); B. Heidtmann (Katholisches Kinderkrankenhaus Wilhelmstift, Germany); S. Toni (AOU Meyer, Italy); B. Rami-Merhar (Medical University of Vienna, Austria); B. Eeckhout, B. Peene, N. Vantongerloo (Algemeen Ziekenhuis Geel Sint-Dimpna Geel, Belgium); T. Maes, L. Gommers (Imeldziekenhuis Bonheiden, Belgium).
