# Supplementary material for: The INNODIA Type 1 Diabetes Natural History Study: a European cohort of newly diagnosed children, adolescents and adults
Source: Diabetologia. 2024 Mar 22;67(6):995–1008. doi: 10.1007/s00125-024-06124-5 (PMC11058619; doi:10.1007/s00125-024-06124-5)

Electronic supplementary material (ESM)

The INNODIA Type 1 Diabetes Natural History Study: a European cohort of newly diagnosed children, adolescents and adults

ML Marcovecchio, AEJ Hendriks, C Delfin, T Battelino, T Danne, ML Evans, J Johannesen, S Kaur, M Knip, L Overbergh, F Pociot, JA Todd, B Van der Schueren, LS Wicker, M Peakman, C Mathieu on behalf of the INNODIA consortium

ESM Table 1. Percentage of autoantibodies type at baseline and 12 months

		**Overall**	**1-9 yr**	**10-17 yr**	**18-45 yr**
**Antibody type positivity at baseline (%)**	IA-2A	70%	76%	69%	54%
	GAD65A	74%	68%	78%	78%
	ZnT8A	67%	68%	71%	52%
	IAA/IA	78%	87%	78%	51%
**Antibody type positivity at 12 months (%)**	IA-2A	68%	68%	72%	56%
	GAD65A	70%	57%	75%	84%
	ZnT8A	61%	53%	69%	55%
	IAA/IA	98%	99%	100%	88%

ESM Fig. 1 Flowchart of study participants

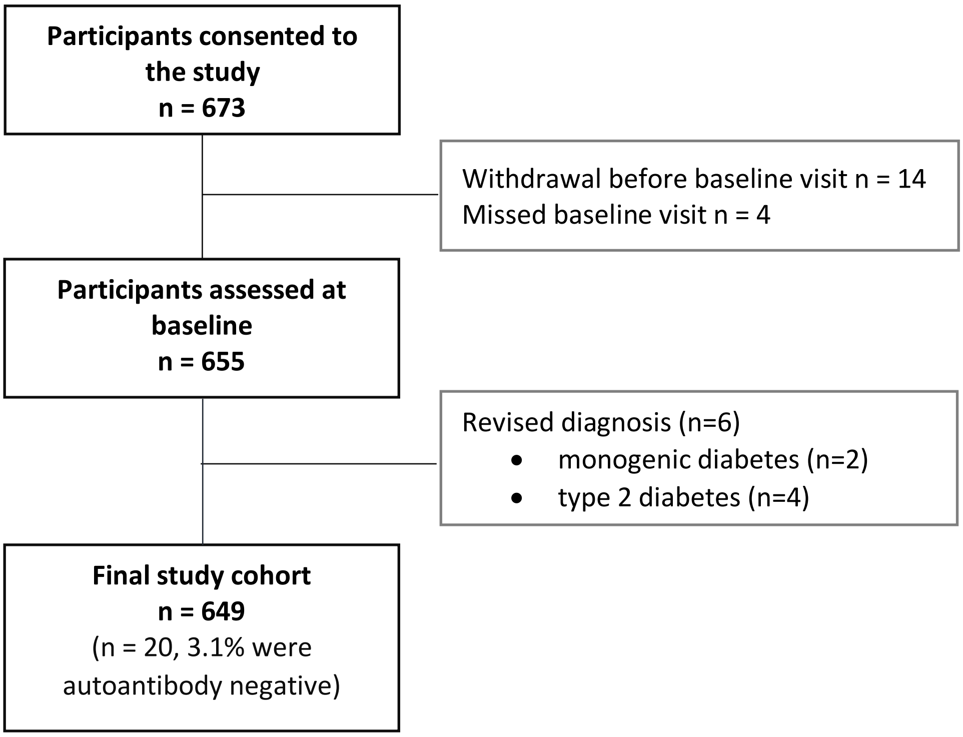

ESM Fig 2. Time course of glucose metabolism parameters over time by sex

Data are reported as median and interquartile range for Fasting C-peptide (a); Fasting glucose (b); AUC C-peptide (c); AUC glucose (d); HbA1c (e); insulin dose (f) and IDAA1c (g).

Green lines represent female and orange lines male

There was no statistically significant difference between males and females in any of the parameters (all p>0.05)

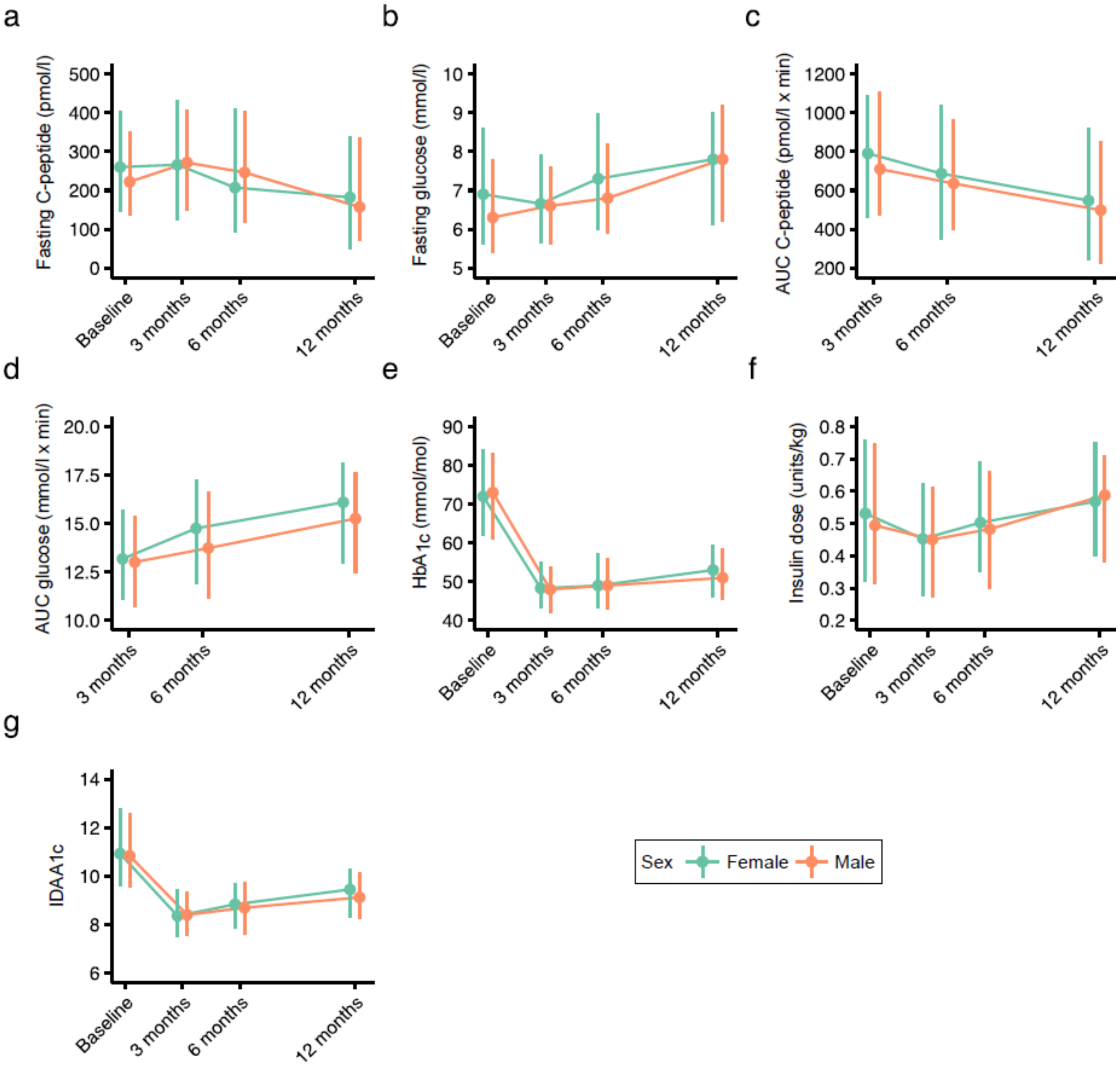

ESM Fig. 3. Time course of glucose metabolism parameters over time by HLA groups
Data are reported as median and interquartile range for Fasting C-peptide (a); Fasting glucose (b); AUC C-peptide (c); AUC glucose (d); HbA1c (e); insulin dose (f) and IDAA1c (g) HLA groups: Group 1 (DR3/DR4; DR4/DR4; DR3/DR3): green lines; Group 2 (DR3/DRX, DR4/DRX): orange lines; Group 3 (DRX/DRX): blue lines.
There was no statistically significant difference between the HLA groups in any of the parameters (all p>0.05)

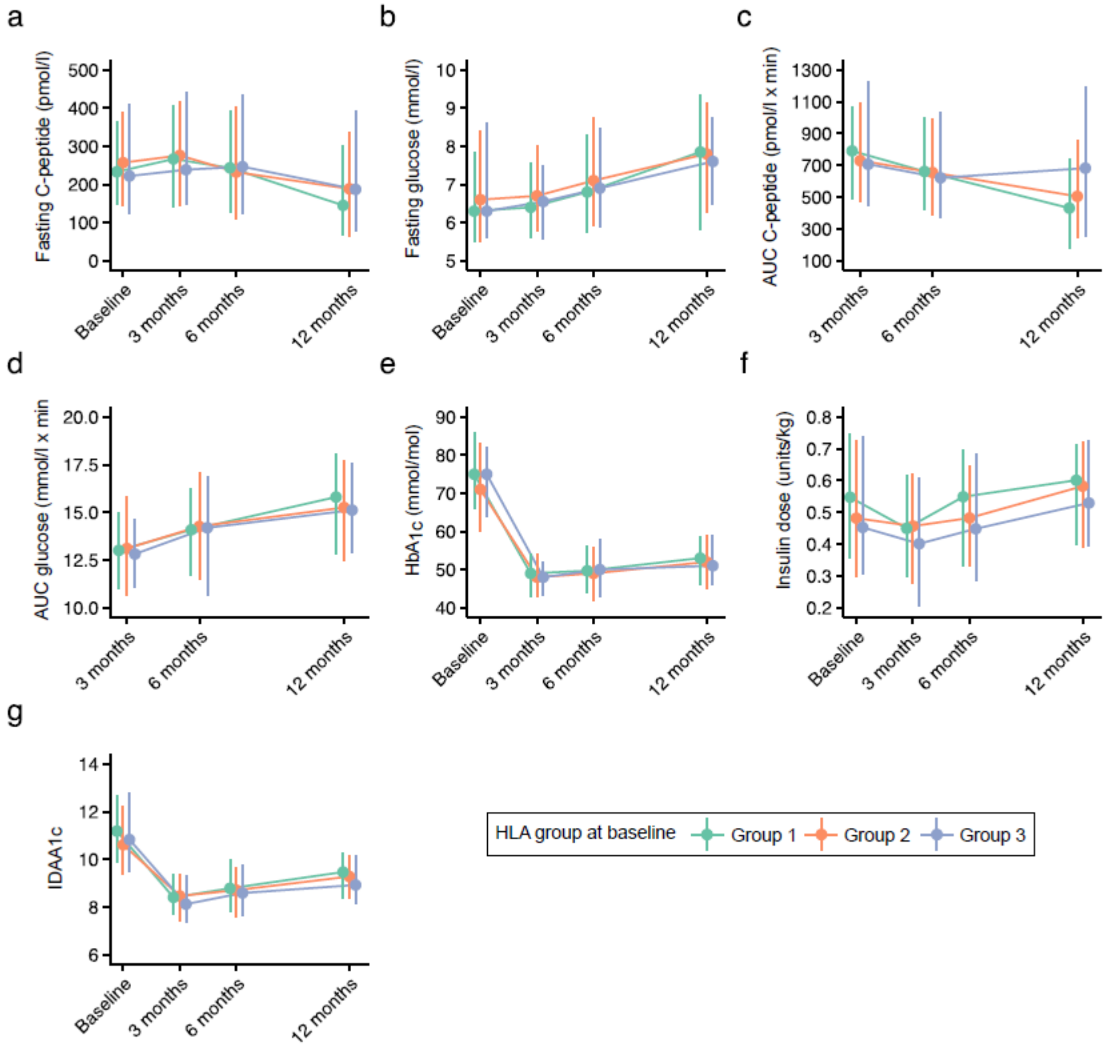

Supplement: Supplementary file 1 — Supplementary file1 (PDF 323 KB) [file 125_2024_6124_MOESM1_ESM.pdf]
